# Their Barefoot Teacher

**Published:** 1892-06

**Authors:** 


### THEIR BAREFOOT TEACHER.

We must adapt our appliances and methods to the work in hand, and we must sacrifice our false notions of dignity to the case, we must save souls, we must pull men out of the fire, we must get hold of them. A Sabbath-school teacher going to his school one afternoon saw a crowd of barefooted boys playing ball. He leaned on the fence and watched them until, growing curious to know why a well-dressed gentleman should be so interested in them, they came up to where he was. He did not break out and scold them for playing ball on the Sabbath, but told them of the ball games he used to play when a boy, and managed to explain that he didn't play on the Sabbath, and why not, that he had a good place to go on Sundays now where lots of other people went. "Say, mister, where is it?" "Well, it is a place where you can go, too." "Where is it?" "It is a Sunday-school." "No, we are barefooted." "Never mind, I will see that you are treated right." "No, we ain't going among a lot of dressed-up kids and we barefooted." "Would you go if your teacher was barefooted, also?" "But he won't be." "But would you?" "Yes, wouldn't we, Bill?" "Of course we would," said Bill, and so said all. Down sat the teacher and off came his shoes and hose. "Now, boys, I am as barefoot as any of you. Come," and they went. That barefooted teacher taught them, notwithstanding the horrified looks of the dilettantes. Well, those boys were back the next Sabbath with shoes on their feet, and out of that class of street arabs so gathered in went forth stalwart Christian men to do heroic work for mankind.

